# Crystal structure of bis­(β-alaninium) tetra­bromidoplumbate

**DOI:** 10.1107/S2056989024007722

**Published:** 2024-08-09

**Authors:** Gayane S. Tonoyan, Gerald Giester, Vahram V. Ghazaryan, Ruben Yu. Chilingaryan, Arthur A. Margaryan, Artak H. Mkrtchyan, Aram M. Petrosyan

**Affiliations:** a Institute of Applied Problems of Physics, NAS of Armenia, 25 Nersessyan Str., 0014 Yerevan, Armenia; b Institute of Mineralogy and Crystallography, University of Vienna, Josef-Holaubek-Platz 2, A-1090 Vienna, Austria; Universidad de la República, Uruguay

**Keywords:** organic–inorganic crystal, hybrid crystal, bromo­plumbate, crystal structure

## Abstract

The title compound, poly[bis­(β-alaninium) [[di­bromido­plumbate]-di-μ-di­bromido]] {(C_2_H_8_NO_2_)_2_[PbBr_4_]}_*n*_ or (*β*-AlaH)_2_PbBr_4_, crystallizes in the monoclinic space group *P*2_1_/*n*. The (PbBr_4_)^2−^ anion is located on a general position and has a two-dimensional polymeric structure. The Pb center is holodirected. The supra­molecular network is mainly based on O—H⋯Br, N—H⋯Br and N—H⋯O hydrogen bonds.

## Abstract 2

This work focused on characterizing the structure from a stereochemical point of view, discussing the Pb^II^ character. The XRD data were collected at 200 K.

## 1. Chemical context

As plumbiferous compounds are commonly toxic, they are unfavorable for photovoltaic devices. Nonetheless, they have other important applications such as white-light-emitting materials (Peng *et al.*, 2018 ▸), luminescent sensing (Wang *et al.*, 2019 ▸; Wang, 2020 ▸; Martínez-Casado *et al.*, 2012 ▸), ferroelectric materials (Gao *et al.*, 2017 ▸), non-linear optical materials (Chen *et al.*, 2020 ▸) and semiconductors (Terpstra *et al.*, 1997 ▸). Lead-containing materials also are attractive from a stereochemical point of view. The Pb^2+^ ion has a 6*s*^2^ electron pair, which is crucial for the stereochemistry of Pb^II^. When the 6*s*^2^ electron pair takes part in hybridization between the *s* and *p* orbitals, the lead atom is stereochemically active and has a hemidirected coordination, otherwise the lead atom exhibits a regular coordination sphere (Casas *et al.*, 2006 ▸; Seth *et al.*, 2018 ▸).

Our research group has been studying various amino acid salts for a long time (Fleck & Petrosyan, 2014 ▸), and we assumed that amino acids could also be used to synthesize organic–inorganic hybrid materials. After the successful synthesis of (GlyH)PbBr_3_ (Tonoyan *et al.*, 2024 ▸), efforts were focused on obtaining (GlyH)PbI_3_ and related phases. Later, it was attempted to synthesize salts of *β*-alanine in the same manner; however, instead of (*β*-AlaH)PbBr_3_, crystals of (*β*-AlaH)_2_PbBr_4_ were formed.
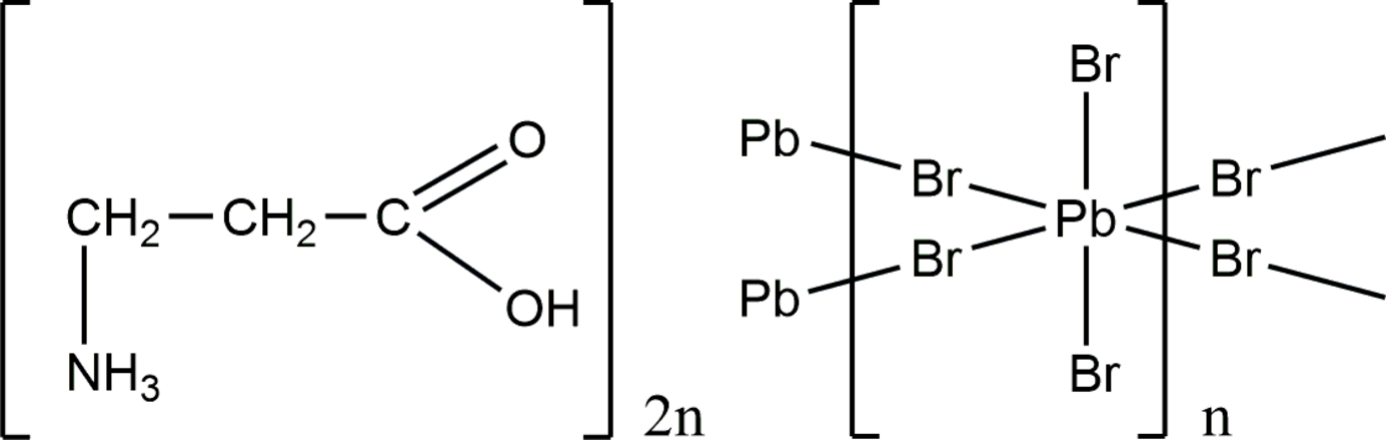


In (*β*-AlaH)_2_PbBr_4_ the anion is slightly distorted and the Pb—Br bond lengths range from 2.8952 (3) to 3.2714 (2) Å. This indicates that the Pb^II^ center is not stereochemically active and the anion has holodirected stereochemistry. Recently a paper was published (Zu *et al.*, 2023 ▸) in which the authors investigated the relationship between the structures and optoelectronic properties of [(HOOC_*n*_H_2*n*−2_NH_3_)_2_]PbBr_4_ (*n* = 3–8) crystals, also including (*β*-AlaH)_2_PbBr_4_ (see also Cazals *et al.*, 2024 ▸). In our work, we focused on characterizing the structure from a stereochemical point of view, discussing the Pb^II^ character in a structure solved using XRD data collected at 200 K.

## 2. Structural commentary

**Figure 1:**
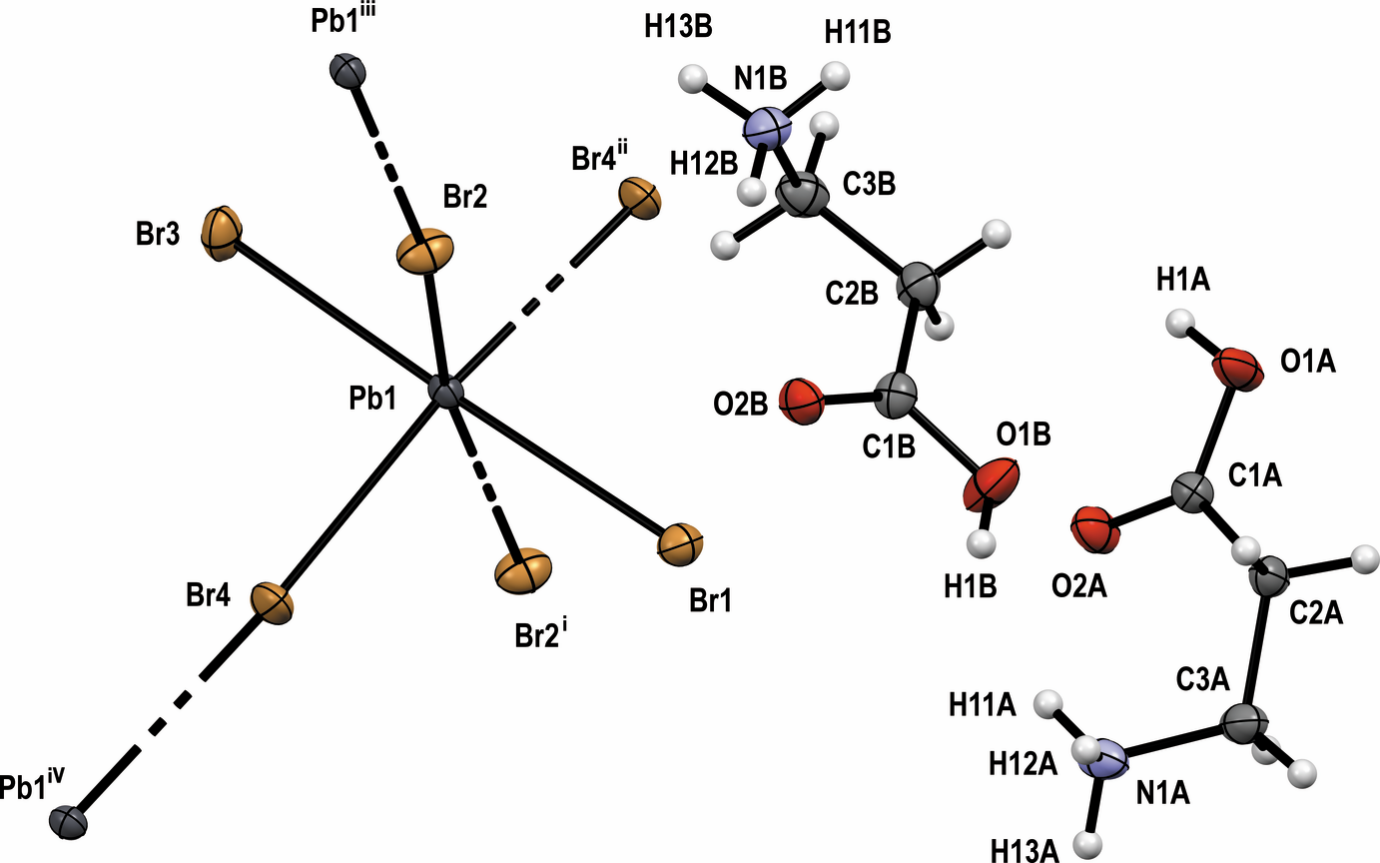
Mol­ecular structure of (*β*-AlaH)_2_PbBr_4_. Displacement ellipsoids are shown at the 50% probability level. Symmetry codes: (i) *x* − 1, *y*, *z*; (ii) −*x* + 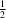, *y* − 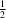, −*z* + 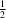; (iii) *x* + 1, *y*, *z*; (iv) −*x* + 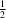, *y* + 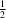, −*z* + 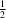.

The title compound (*β*-AlaH)_2_PbBr_4_ crystallizes in the monoclinic space group *P*2_1_/*n*. The asymmetric unit contains one formula unit. The mol­ecular arrangement is shown in Fig. 1 ▸. As can be seen from the dihedral angles (Table 1 ▸), both *β*-alaninium cations have the most common *gauche* conformation (Fleck *et al.*, 2012 ▸).

**Figure 2:**
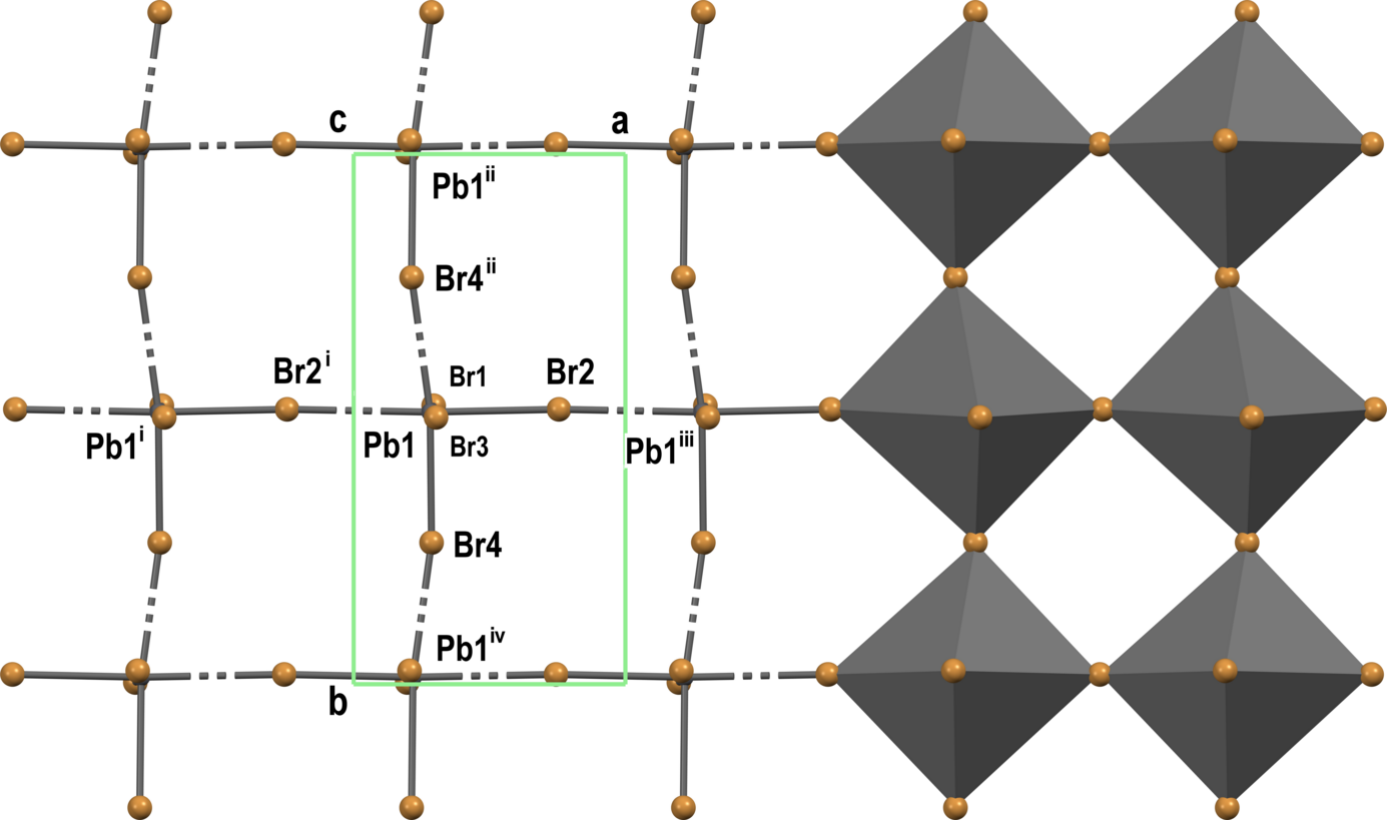
2D structure of the PbBr_4_ anion viewed along the *c* axis. Part of the anion is shown in an octa­hedral style. Symmetry codes: (i) *x* − 1, *y*, *z*; (ii) −*x* + 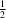, *y* − 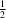, −*z* + 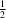; (iii) *x* + 1, *y*, *z*; (iv) −*x* + 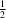, *y* + 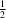, −*z* + 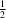.

The Pb^2+^ centers of the anion exhibit a holodirected six-coordination with an octa­hedral geometry. Therefore, for neighboring bromine atoms, the Br—Pb—Br angles are close to right angles, varying from 80.90 (1) to 103.14 (1)° (Table 1 ▸). The lead atom forms three partial covalent bonds Pb1—Br2, Pb1—Br3, and Pb1—Br4, and also three coordination bonds with partial covalent character Pb1—Br1, Pb1—Br2^i^ and Pb1—Br4^ii^ (Table 1 ▸). Despite the range of Pb1—Br distances, the average value of 3.0259 Å is close to the average value of 3.0310 Å in PbBr_6_ octa­hedra, regardless of the anion, for 284 structures in the Cambridge Structural Database (CSD2023.2.0, version 5.45, November update; Groom *et al.*, 2016 ▸). The PbBr_6_ octa­hedra form a 2D structure with four shared vertices: Br2, Br2^i^, Br4, and Br4^ii^ (Fig. 2 ▸). The octa­hedra share only vertices, not edges nor faces. The two terminal opposite atoms Br1 and Br3 are located on the surfaces of the layer and the octa­hedra are arranged in such a way that the angles of the Pb—Br—Pb bridges are close to linear (Table 1 ▸), which leads to square-shaped voids between the octa­hedra.

## 3. Supra­molecular features

**Figure 3:**
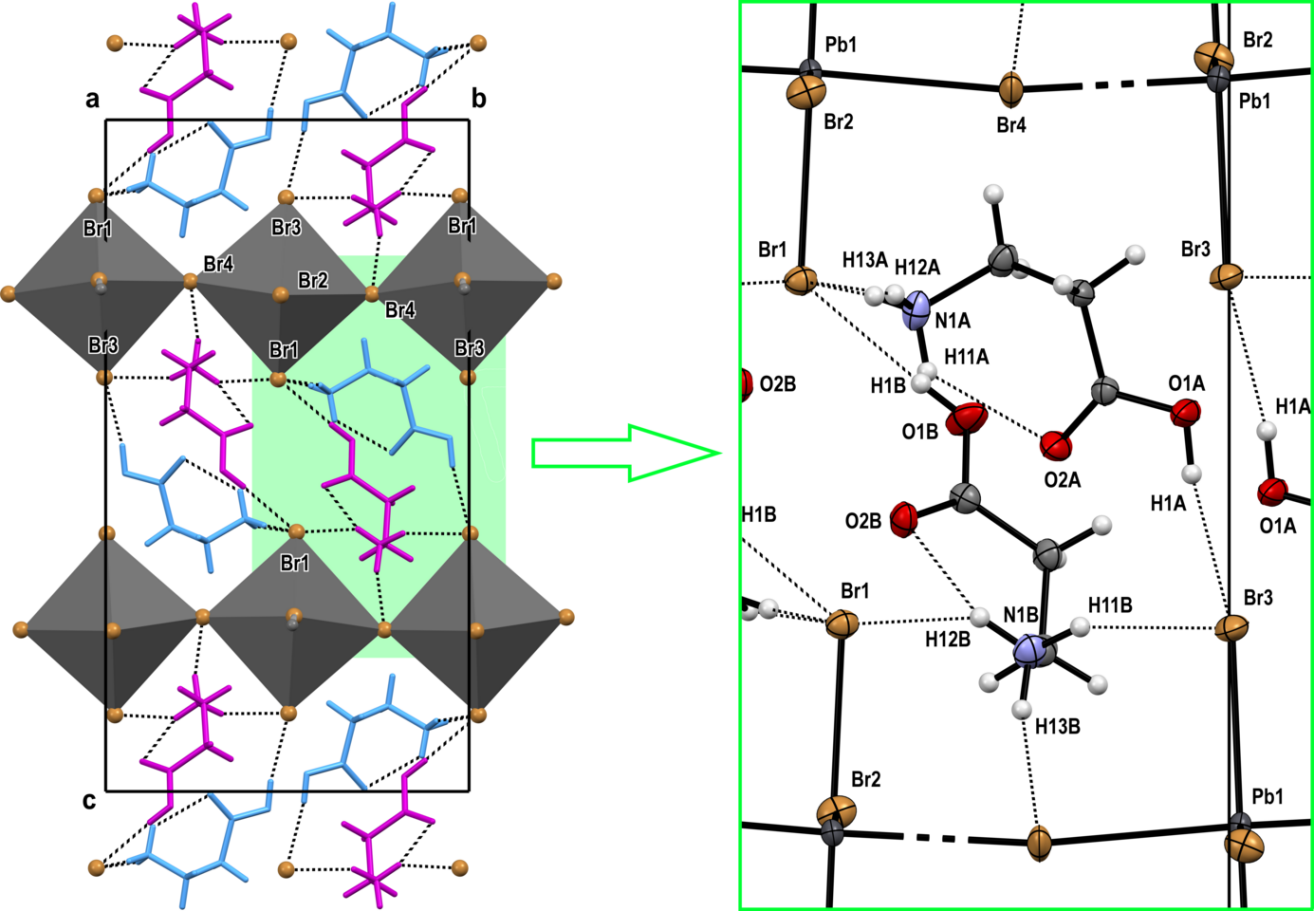
Packing diagram of the structure of (*β*-AlaH)_2_PbBr_4_ viewed along the *a* axis. Hydrogen bonds are shown as dotted lines.

The packing in the crystal together with the hydrogen-bond network is shown in Fig. 3 ▸. The anionic layers are parallel to the (001) plane, with an inter­layer distance of 11.026 (1) Å. The *β*-alaninium cations are positioned between the anionic layers with the amino and carboxyl groups oriented towards those layers. The *β*-alaninium cations cross-link neighboring layers of anions through hydrogen bonds between terminal bromine atoms and NH_3_^+^, and OH groups (Table 2 ▸). Each carboxyl group forms one O—H⋯Br hydrogen bond, while the ammonium groups form two and three N—H⋯Br hydrogen bonds. Intra­molecular N1*A*—H11*A*⋯O2*A* and N1*B*—H12*B*⋯O2*B* hydrogen bonds are present in the *β*-alaninium moieties (Table 2 ▸).

## 4. Database survey

A survey of the Cambridge Structural Database (CSD2023.2.0, version 5.45, November update; Groom *et al.*, 2016 ▸) revealed 320 structures containing PbBr_4_. There were 91 duplicate structures solved at different temperatures, and several inappropriate structures; thus, overall 224 structures were considered. Among them, the title compound was found with refcode YINFIO (Zu *et al.*, 2023 ▸), determined at room temperature.

**Figure 4:**
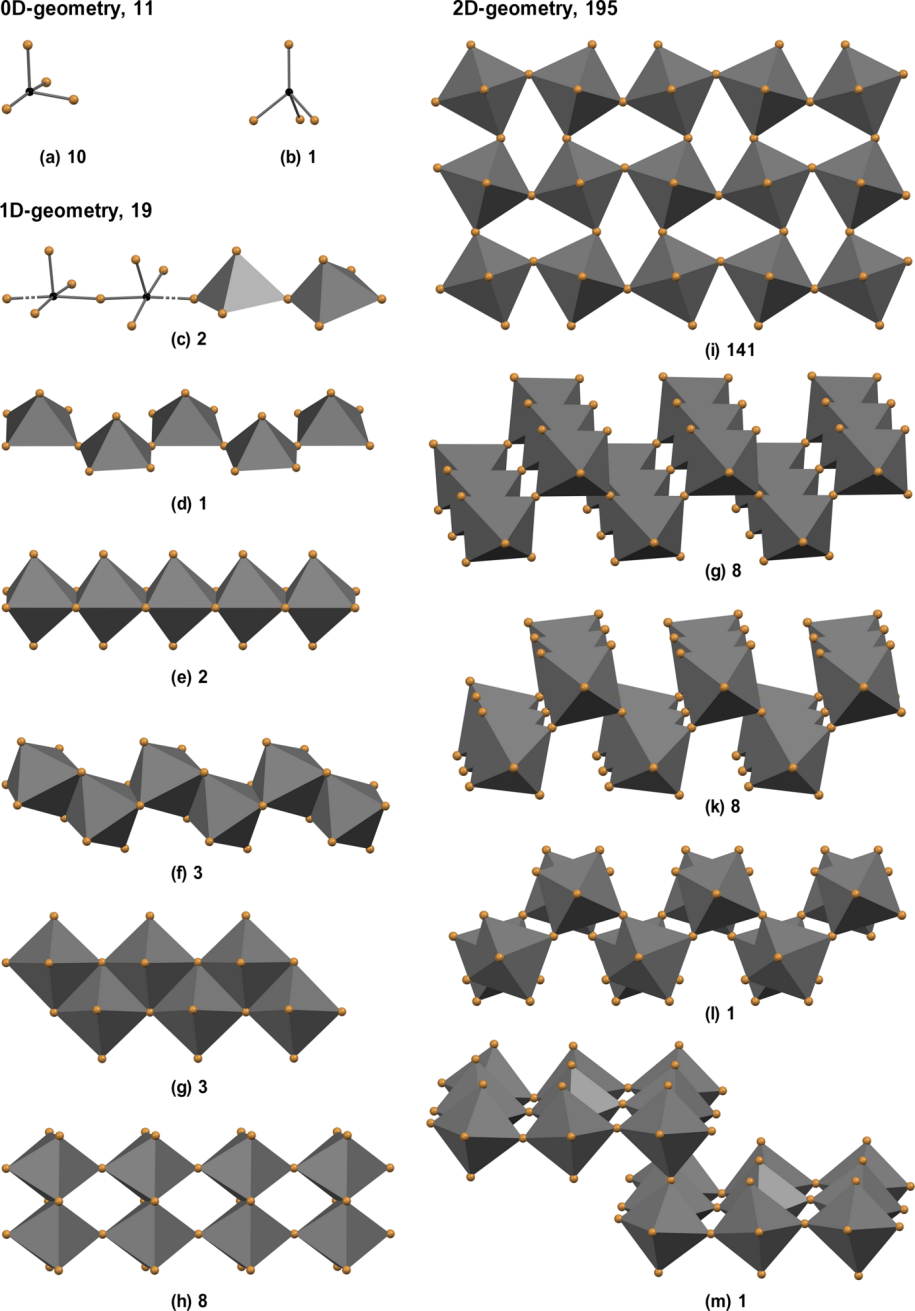
The (PbBr_4_)^2−^ anion geometries. Note that one more 2D form is missing here as it is shown in Fig. 2 ▸. The numbers of CCD structures for a given type of anion are indicated.

In the structures, the (PbBr_4_)^2−^ anion can be in discrete 0D, polymeric 1D, and 2D forms. 0D anions are present in a *pseudotrigonal–bipyramidal geometry* (Fig. 4 ▸*a*: ARAJUB, Lin *et al.*, 2019 ▸; BOKYAF, Han 2024 ▸; YIQPAP, Gröger *et al.*, 2002 ▸) and in a *trigonal–pyramidal* geometry (Fig. 4 ▸*b*: UVELIT, Gong *et al.*, 2021 ▸).

A 1D structure anion may consist of either PbBr_5_ square-pyramids (3 structures) or PbBr_6_ octa­hedra (16 structures). The square-pyramids are alternately connected by a shared bromine atom, with three bromine atoms remaining terminal. Chains can be *linear* (Fig. 4 ▸*c*: RUSBUF, Lv *et al.*, 2020 ▸) or *zigzag* (Fig. 4 ▸*d*: SOHYAS, Li *et al.*, 2019 ▸). Octa­hedral PbBr_6_ monomers can attach two, three or four adjacent octa­hedra, have four or three shared bromine atoms, and two or three terminal atoms. Chains can be *linear* (Fig. 4 ▸*e*: COKYIO, Zhang *et al.*, 2024 ▸), *zigzag* (Fig. 4 ▸*f*: CEKYIE, Fu *et al.*, 2022 ▸), *V-shaped* (Fig. 4 ▸*g*: FERGER, Yuan *et al.*, 2017 ▸), and *double* (Fig. 4 ▸*h*: HENLAR, Jin *et al.*, 2022 ▸).

When each monomer has four adjacent monomers attached, and each pair of adjacent monomers shares one bromine atom, a 2D structure is formed (195 structures). Each lead atom has two terminal bromine atoms in the 2D structure of the anion. There are two main options, depending on the terminal atoms. When terminal atoms are *trans* positioned, a planar arrangement of octa­hedra is formed. In our case, the Pb—Br—Pb angles are close to linear (Table 1 ▸, Fig. 2 ▸). An ideal form of this is a rare centrosymmetric anion in the structure of COJKIZ01 (Long *et al.*, 2024 ▸) with 180° Pb—Br—Pb angles, and *square*-shaped voids between the octa­hedra. There are 36 structures with square or near square voids, and this is the second most common geometry at 16%. In other cases, the Pb—Br—Pb angles differ from 180°, and values down to 139° can be encountered, causing *rhombic* voids (Fig. 4 ▸*i*: TAKZAK, Zhang, *et al.*, 2020 ▸; OBAYAV, Zhang *et al.*, 2021 ▸). This geometry is found in the prevailing number of structures, 141, almost 63%. However, in the case of *cis* positioning of terminal atoms, the anion has a zigzag arrangement of octa­hedra, forming stacked layers. Two-stacked layers can be formed having linear Pb—Br—Pb angles (Fig. 4 ▸*g:* SOHYAS01, Li *et al.*, 2019 ▸) or obtuse angles in the range of 164–148° (Fig. 4 ▸*k*: RICBEO01, RICBEO02, Drozdowski *et al.*, 2023 ▸; Fig. 4 ▸*l*: NIZQAP, Li *et al.*, 2008 ▸). One structure has layers arranged in a zigzag manner that contain octa­hedra with both *trans*- and *cis*-positioned terminal bromine atoms (Fig. 4 ▸*m*: UBUFEG, Guo *et al.*, 2021 ▸).

## 5. Synthesis and crystallization

**Figure 5:**
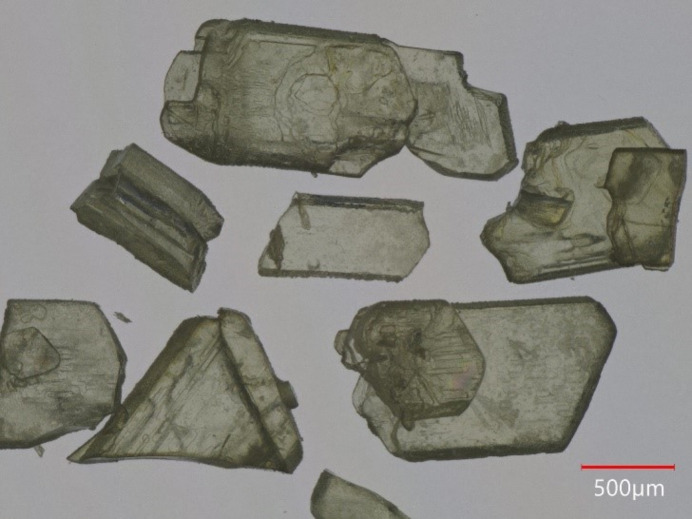
Yellow crystals of (*β*-AlaH)_2_PbBr_4_ under the microscope.

As initial reagents, we used amino acid *β*-alanine (99% NT) and hydro­bromic acid (48%) purchased from Sigma-Aldrich and lead (reactive grade). Initially, an excess volume of hydro­bromic acid was added to a preliminary weighted amount of lead. When the reaction between them was completed (when no H_2_ gas is released), the unreacted lead was removed by filtration, dried and weighed. The qu­anti­ties of lead(II) bromide (PbBr_2_) obtained and unreacted acid (HBr) in the filtrate were calculated. The appropriate amount of *β*-alanine was added to it and mixed to achieve a final solution with a 1:1:6 molar ratio of *β*-Ala, PbBr_2_ and HBr, respectively. Instead of the desired compound (*β*-AlaH)PbBr_3_, (*β*-AlaH)_2_PbBr_4_ was obtained as yellow crystals (Fig. 5 ▸).

## 6. Refinement

Crystal data, data collection and structure refinement details are summarized in Table 3 ▸. Hydrogen atoms were treated as riding on their parent atoms [C—H = 0.99 Å, N—H = 0.91 Å; *U*_iso_(H) = 1.2U_eq_(C) or *U*_iso_(H) = 1.5U_eq_(N)] except those of the carboxyl groups, which were refined with the restraint *U*_iso_(H) = 1.5U_eq_(C).

## Supplementary Material

Crystal structure: contains datablock(s) I. DOI: 10.1107/S2056989024007722/oo2006sup1.cif

Structure factors: contains datablock(s) I. DOI: 10.1107/S2056989024007722/oo2006Isup2.hkl

Supporting information file. DOI: 10.1107/S2056989024007722/oo2006Isup3.mol

CCDC reference: 2368897

Additional supporting information:  crystallographic information; 3D view; checkCIF report

## Figures and Tables

**Table 1:** Selected geometric parameters (Å, °)

Pb1—Br1	3.0589 (4)	Pb1—Br2^i^	3.2714 (2)
Pb1—Br3	2.9230 (4)	Pb1—Br4	2.9477 (3)
Pb1—Br2	2.8952 (3)	Pb1—Br4^ii^	3.0591 (3)
			
Br1—Pb1—Br2	88.107 (9)	Br2^i^—Pb1—Br4^ii^	81.63 (1)
Br1—Pb1—Br2^i^	80.90 (1)	Br3—Pb1—Br2	87.836 (9)
Br1—Pb1—Br4	87.683 (8)	Br3—Pb1—Br4	92.596 (8)
Br1—Pb1—Br4^ii^	89.760 (8)	Br3—Pb1—Br4^ii^	90.472 (8)
Br2—Pb1—Br4	90.568 (9)	Pb1—Br2—Pb1^iii^	168.87 (1)
Br2—Pb1—Br4^ii^	96.645 (9)	Pb1—Br2^i^—Pb1^i^	168.87 (1)
Br2^i^—Pb1—Br4	90.75 (1)	Pb1—Br4—Pb1^iv^	168.90 (1)
			
O1*A*—C1*A*—C2*A*—C3a	−164.4 (2)	O1*B*—C1*B*—C2*B*—C3*B*	171.4 (2)
C1*A*—C2*A*—C3*A*—N1*A*	−62.9 (3)	C1*B*—C2*B*—C3*B*—N1*B*	59.7 (3)

Symmetry codes: (i) 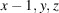; (ii) 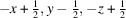; (iii) 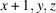; (iv) 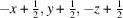.

**Table 2:** Hydrogen-bond geometry (Å, °)

*D*—H⋯*A*	*D*—H	H⋯*A*	*D*⋯*A*	*D*—H⋯*A*
O1*A*—H1*A*⋯Br3^ii^	0.90 (2)	2.35 (3)	3.241 (2)	171 (4)
N1*A*—H11*A*⋯O2*A*	0.91	2.18	2.853 (3)	130
N1*A*—H12*A*⋯Br1^v^	0.91	2.73	3.619 (2)	166
N1*A*—H13*A*⋯Br1^vi^	0.91	2.58	3.445 (2)	159
O1*B*—H1*B*⋯Br1^v^	0.88 (2)	2.32 (3)	3.188 (2)	167 (4)
N1*B*—H11*B*⋯Br3^vii^	0.91	2.49	3.343 (2)	157
N1*B*—H12*B*⋯Br1^iii^	0.91	2.76	3.484 (2)	137
N1*B*—H12*B*⋯O2*B*	0.91	2.17	2.839 (3)	129
N1*B*—H13*B*⋯Br4^vii^	0.91	2.56	3.407 (2)	155

Symmetry codes: (ii) 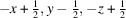; (iii) 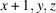; (v) 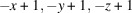; (vi) 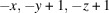; (vii) 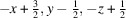.

**Table 3:** Experimental details

Crystal data	
Chemical formula	(C_3_H_8_NO_2_)_2_[PbBr_4_]
*M*_r_	707.04
Crystal system, space group	Monoclinic, *P*2_1_/*n*
Temperature (K)	200
*a*, *b*, *c* (Å)	6.1377 (4), 11.9291 (8), 22.1508 (14)
β (°)	95.402 (2)
*V* (Å^3^)	1614.62 (18)
*Z*	4
Radiation type	Mo *K*α
μ (mm^−1^)	20.35
Crystal size (mm)	0.20 × 0.18 × 0.08

Data collection	
Diffractometer	Bruker APEXII CCD
Absorption correction	Multi-scan (*SADABS*; Krause *et al.*, 2015 ▸)
*T*_min_, *T*_max_	0.346, 0.747
No. of measured, independent and observed [*I* > 2σ(*I*)] reflections	53070, 6172, 5328
*R*_int_	0.046
(sin θ/λ)_max_ (Å^−1^)	0.770

Refinement	
*R*[*F*^2^ > 2σ(*F*^2^)], *wR*(*F*^2^), *S*	0.023, 0.044, 1.07
No. of reflections	6172
No. of parameters	163
No. of restraints	2
H-atom treatment	H atoms treated by a mixture of independent and constrained refinement
Δρ_max_, Δρ_min_ (e Å^−3^)	1.51, −1.16

Computer programs: *APEX5* Bruker (2024 ▸), *SHELXT* (Sheldrick, 2015*a* ▸), *SHELXL2019/2* (Sheldrick, 2015*b* ▸) and *ShelXle* (Hübschle *et al.*, 2011 ▸).

## supplementary crystallographic information

### Poly[bis(β-alaninium) [[dibromidoplumbate]-di-µ-dibromido]] Crystal data

**Table:**

(C_3_H_8_NO_2_)_2_[PbBr_4_]	*F*(000) = 1280
*M**_r_* = 707.04	*D*_x_ = 2.909 Mg m^−^^3^
Monoclinic, *P*2_1_/*n*	Mo *K*α radiation, λ = 0.71073 Å
*a* = 6.1377 (4) Å	Cell parameters from 9846 reflections
*b* = 11.9291 (8) Å	θ = 3.4–32.8°
*c* = 22.1508 (14) Å	µ = 20.35 mm^−^^1^
β = 95.402 (2)°	*T* = 200 K
*V* = 1614.62 (18) Å^3^	Plate, yellow
*Z* = 4	0.20 × 0.18 × 0.08 mm

### Poly[bis(β-alaninium) [[dibromidoplumbate]-di-µ-dibromido]] Data collection

**Table:**

Bruker APEXII CCD diffractometer	5328 reflections with *I* > 2σ(*I*)
φ and ω scans	*R*_int_ = 0.046
Absorption correction: multi-scan (SADABS; Krause *et al.*, 2015)	θ_max_ = 33.2°, θ_min_ = 1.9°
*T*_min_ = 0.346, *T*_max_ = 0.747	*h* = −9→9
53070 measured reflections	*k* = −18→18
6172 independent reflections	*l* = −33→33

### Poly[bis(β-alaninium) [[dibromidoplumbate]-di-µ-dibromido]] Refinement

**Table:**

Refinement on *F*^2^	Secondary atom site location: difference Fourier map
Least-squares matrix: full	Hydrogen site location: mixed
*R*[*F*^2^ > 2σ(*F*^2^)] = 0.023	H atoms treated by a mixture of independent and constrained refinement
*wR*(*F*^2^) = 0.044	*w* = 1/[σ^2^(*F*_o_^2^) + (0.0092*P*)^2^ + 2.110*P*] where *P* = (*F*_o_^2^ + 2*F*_c_^2^)/3
*S* = 1.07	(Δ/σ)_max_ = 0.003
6172 reflections	Δρ_max_ = 1.51 e Å^−^^3^
163 parameters	Δρ_min_ = −1.16 e Å^−^^3^
2 restraints	Extinction correction: *SHELXL2019/2* (Sheldrick, 2015b), Fc^*^=kFc[1+0.001xFc^2^λ^3^/sin(2θ)]^-1/4^
Primary atom site location: structure-invariant direct methods	Extinction coefficient: 0.00134 (4)

### Poly[bis(β-alaninium) [[dibromidoplumbate]-di-µ-dibromido]] Special details

**Table:**

Experimental. A selected fragment of a crystal was mounted on a *MiTeGen* loop with silicone grease and examined by single crystal X-ray diffraction at 200 K on a Bruker APEX II diffractometer equipped with a CCD area detector, an Incoatec Microfocus Source IµS (30 W, multilayer mirror, Mo-K_α_) and an Oxford Cryosystems Cryostream 800 Plus LT device. Several sets of phi- and omega-scans with 2° scanwidth were combined at a crystal-detector distances of 40 mm to achieve respective full sphere data up to 65° 2θ. Data handling with integration and absorption correction by evaluation of multi-scans was done with the Bruker Apex5 suite (Bruker, 2024). The structure was solved by direct methods (Sheldrick, 2015a); subsequent difference Fourier syntheses and least-squares refinements yielded the positions of the remaining atoms using the SHELX software (Sheldrick, 2015b) implemented in the ShelXle GUI tool (Hübschle *et al.* 2011). Non-hydrogen atoms were refined with independent anisotropic displacement parameters.
Geometry. All esds (except the esd in the dihedral angle between two l.s. planes) are estimated using the full covariance matrix. The cell esds are taken into account individually in the estimation of esds in distances, angles and torsion angles; correlations between esds in cell parameters are only used when they are defined by crystal symmetry. An approximate (isotropic) treatment of cell esds is used for estimating esds involving l.s. planes.

### Poly[bis(β-alaninium) [[dibromidoplumbate]-di-µ-dibromido]] Fractional atomic coordinates and isotropic or equivalent isotropic displacement parameters (Å^2^)

**Table:**

	*x*	*y*	*z*	*U*_iso_*/*U*_eq_	
Pb1	0.28160 (2)	0.48657 (2)	0.24772 (2)	0.01445 (3)	
Br1	0.29433 (4)	0.47456 (2)	0.38595 (2)	0.02132 (6)	
Br2	0.75530 (4)	0.48144 (3)	0.26094 (2)	0.02559 (6)	
Br3	0.30325 (5)	0.49665 (2)	0.11657 (2)	0.02542 (6)	
Br4	0.28669 (5)	0.73272 (2)	0.25942 (2)	0.02607 (7)	
O1A	0.3047 (4)	0.05338 (17)	0.52622 (10)	0.0313 (5)	
H1A	0.278 (6)	0.046 (3)	0.4858 (11)	0.047*	
O2A	0.1232 (3)	0.21235 (17)	0.50427 (9)	0.0269 (4)	
C1A	0.2260 (4)	0.1520 (2)	0.54084 (12)	0.0186 (5)	
C2A	0.2849 (5)	0.1821 (2)	0.60573 (12)	0.0200 (5)	
H21A	0.441345	0.203553	0.611180	0.024*	
H22A	0.265866	0.115205	0.631189	0.024*	
C3A	0.1492 (5)	0.2771 (2)	0.62751 (14)	0.0239 (6)	
H31A	−0.007460	0.256173	0.621905	0.029*	
H32A	0.189159	0.289100	0.671382	0.029*	
N1A	0.1826 (4)	0.38366 (19)	0.59418 (12)	0.0258 (5)	
H11A	0.166295	0.370391	0.553548	0.039*	
H12A	0.319688	0.410268	0.604972	0.039*	
H13A	0.082103	0.435237	0.603713	0.039*	
O1B	0.6103 (4)	0.3217 (2)	0.52220 (10)	0.0340 (5)	
H1B	0.658 (6)	0.378 (3)	0.5458 (16)	0.051*	
O2B	0.8138 (3)	0.39905 (15)	0.45540 (9)	0.0235 (4)	
C1B	0.6963 (4)	0.3238 (2)	0.46965 (13)	0.0204 (5)	
C2B	0.6369 (5)	0.2227 (2)	0.43163 (13)	0.0221 (5)	
H21B	0.476516	0.211567	0.429550	0.026*	
H22B	0.707312	0.156012	0.451675	0.026*	
C3B	0.7035 (5)	0.2303 (2)	0.36840 (14)	0.0263 (6)	
H31B	0.657467	0.161277	0.345882	0.032*	
H32B	0.627841	0.294589	0.347349	0.032*	
N1B	0.9441 (4)	0.2447 (2)	0.36811 (12)	0.0269 (5)	
H11B	1.013572	0.182729	0.384263	0.040*	
H12B	0.987954	0.305927	0.390577	0.040*	
H13B	0.977876	0.254316	0.329319	0.040*	

### Poly[bis(β-alaninium) [[dibromidoplumbate]-di-µ-dibromido]] Atomic displacement parameters (Å^2^)

**Table:**

	*U*^11^	*U*^22^	*U*^33^	*U*^12^	*U*^13^	*U*^23^
Pb1	0.01638 (5)	0.01111 (4)	0.01587 (5)	−0.00006 (3)	0.00157 (3)	0.00083 (3)
Br1	0.02060 (13)	0.02395 (12)	0.01946 (14)	0.00223 (9)	0.00222 (10)	−0.00132 (10)
Br2	0.01714 (12)	0.03413 (15)	0.02531 (15)	0.00159 (10)	0.00106 (11)	−0.00382 (11)
Br3	0.03326 (16)	0.02572 (13)	0.01649 (13)	−0.00212 (11)	−0.00183 (11)	0.00417 (10)
Br4	0.03725 (16)	0.01262 (11)	0.02997 (16)	−0.00035 (10)	0.01171 (12)	−0.00028 (10)
O1A	0.0530 (15)	0.0219 (10)	0.0179 (11)	0.0140 (9)	−0.0025 (10)	−0.0030 (8)
O2A	0.0340 (12)	0.0245 (10)	0.0209 (11)	0.0097 (8)	−0.0045 (9)	0.0011 (8)
C1A	0.0201 (13)	0.0166 (11)	0.0192 (13)	0.0010 (9)	0.0026 (10)	0.0001 (9)
C2A	0.0255 (14)	0.0184 (11)	0.0157 (13)	0.0034 (10)	0.0002 (10)	0.0002 (9)
C3A	0.0269 (14)	0.0203 (12)	0.0255 (15)	0.0000 (10)	0.0086 (12)	−0.0038 (11)
N1A	0.0255 (12)	0.0179 (10)	0.0350 (15)	0.0002 (9)	0.0082 (11)	−0.0043 (10)
O1B	0.0397 (13)	0.0392 (13)	0.0254 (12)	−0.0156 (10)	0.0152 (10)	−0.0090 (9)
O2B	0.0255 (10)	0.0172 (9)	0.0287 (11)	−0.0041 (7)	0.0066 (9)	−0.0009 (8)
C1B	0.0184 (13)	0.0221 (12)	0.0206 (14)	−0.0014 (9)	0.0013 (10)	0.0012 (10)
C2B	0.0222 (13)	0.0199 (12)	0.0244 (15)	−0.0052 (10)	0.0037 (11)	−0.0004 (10)
C3B	0.0283 (15)	0.0244 (13)	0.0254 (15)	0.0026 (11)	−0.0013 (12)	−0.0033 (11)
N1B	0.0305 (13)	0.0235 (11)	0.0283 (14)	0.0017 (10)	0.0111 (11)	−0.0014 (10)

### Poly[bis(β-alaninium) [[dibromidoplumbate]-di-µ-dibromido]] Geometric parameters (Å, º)

**Table:**

Pb1—Br1	3.0589 (4)	N1A—H11A	0.9100
Pb1—Br3	2.9230 (4)	N1A—H12A	0.9100
Pb1—Br2	2.8952 (3)	N1A—H13A	0.9100
Pb1—Br2^i^	3.2714 (2)	O1B—C1B	1.323 (3)
Pb1—Br4	2.9477 (3)	O1B—H1B	0.88 (2)
Pb1—Br4^ii^	3.0591 (3)	O2B—C1B	1.212 (3)
O1A—C1A	1.323 (3)	C1B—C2B	1.497 (4)
O1A—H1A	0.90 (2)	C2B—C3B	1.498 (4)
O2A—C1A	1.215 (3)	C2B—H21B	0.9900
C1A—C2A	1.493 (4)	C2B—H22B	0.9900
C2A—C3A	1.512 (4)	C3B—N1B	1.487 (4)
C2A—H21A	0.9900	C3B—H31B	0.9900
C2A—H22A	0.9900	C3B—H32B	0.9900
C3A—N1A	1.494 (4)	N1B—H11B	0.9100
C3A—H31A	0.9900	N1B—H12B	0.9100
C3A—H32A	0.9900	N1B—H13B	0.9100
			
Br1—Pb1—Br2	88.107 (9)	C2A—C3A—H32A	109.2
Br1—Pb1—Br2^i^	80.90 (1)	H31A—C3A—H32A	107.9
Br1—Pb1—Br4	87.683 (8)	C3A—N1A—H11A	109.5
Br1—Pb1—Br4^ii^	89.760 (8)	C3A—N1A—H12A	109.5
Br2—Pb1—Br4	90.568 (9)	H11A—N1A—H12A	109.5
Br2—Pb1—Br4^ii^	96.645 (9)	C3A—N1A—H13A	109.5
Br2^i^—Pb1—Br4	90.75 (1)	H11A—N1A—H13A	109.5
Br2^i^—Pb1—Br4^ii^	81.63 (1)	H12A—N1A—H13A	109.5
Br3—Pb1—Br2	87.836 (9)	C1B—O1B—H1B	112 (3)
Br3—Pb1—Br4	92.596 (8)	O2B—C1B—O1B	122.7 (3)
Br3—Pb1—Br4^ii^	90.472 (8)	O2B—C1B—C2B	124.7 (3)
Pb1—Br2—Pb1^iii^	168.87 (1)	O1B—C1B—C2B	112.5 (2)
Pb1—Br2^i^—Pb1^i^	168.87 (1)	C1B—C2B—C3B	113.8 (2)
Pb1—Br4—Pb1^iv^	168.90 (1)	C1B—C2B—H21B	108.8
Br3—Pb1—Br1	175.936 (9)	C3B—C2B—H21B	108.8
Br4—Pb1—Br4^ii^	172.266 (4)	C1B—C2B—H22B	108.8
C1A—O1A—H1A	107 (3)	C3B—C2B—H22B	108.8
O2A—C1A—O1A	122.8 (3)	H21B—C2B—H22B	107.7
O2A—C1A—C2A	124.2 (2)	N1B—C3B—C2B	111.7 (2)
O1A—C1A—C2A	112.9 (2)	N1B—C3B—H31B	109.3
C1A—C2A—C3A	113.4 (2)	C2B—C3B—H31B	109.3
C1A—C2A—H21A	108.9	N1B—C3B—H32B	109.3
C3A—C2A—H21A	108.9	C2B—C3B—H32B	109.3
C1A—C2A—H22A	108.9	H31B—C3B—H32B	107.9
C3A—C2A—H22A	108.9	C3B—N1B—H11B	109.5
H21A—C2A—H22A	107.7	C3B—N1B—H12B	109.5
N1A—C3A—C2A	112.0 (2)	H11B—N1B—H12B	109.5
N1A—C3A—H31A	109.2	C3B—N1B—H13B	109.5
C2A—C3A—H31A	109.2	H11B—N1B—H13B	109.5
N1A—C3A—H32A	109.2	H12B—N1B—H13B	109.5
			
O1A—C1A—C2A—C3a	−164.4 (2)	O1B—C1B—C2B—C3B	171.4 (2)
C1A—C2A—C3A—N1A	−62.9 (3)	C1B—C2B—C3B—N1B	59.7 (3)

Symmetry codes: (i) *x*−1, *y*, *z*; (ii) −*x*+1/2, *y*−1/2, −*z*+1/2; (iii) *x*+1, *y*, *z*; (iv) −*x*+1/2, *y*+1/2, −*z*+1/2.

### Poly[bis(β-alaninium) [[dibromidoplumbate]-di-µ-dibromido]] Hydrogen-bond geometry (Å, º)

**Table:**

*D*—H···*A*	*D*—H	H···*A*	*D*···*A*	*D*—H···*A*
O1*A*—H1*A*···Br3^ii^	0.90 (2)	2.35 (3)	3.241 (2)	171 (4)
N1*A*—H11*A*···O2*A*	0.91	2.18	2.853 (3)	130
N1*A*—H12*A*···Br1^v^	0.91	2.73	3.619 (2)	166
N1*A*—H13*A*···Br1^vi^	0.91	2.58	3.445 (2)	159
O1*B*—H1*B*···Br1^v^	0.88 (2)	2.32 (3)	3.188 (2)	167 (4)
N1*B*—H11*B*···Br3^vii^	0.91	2.49	3.343 (2)	157
N1*B*—H12*B*···Br1^iii^	0.91	2.76	3.484 (2)	137
N1*B*—H12*B*···O2*B*	0.91	2.17	2.839 (3)	129
N1*B*—H13*B*···Br4^vii^	0.91	2.56	3.407 (2)	155

Symmetry codes: (ii) −*x*+1/2, *y*−1/2, −*z*+1/2; (iii) *x*+1, *y*, *z*; (v) −*x*+1, −*y*+1, −*z*+1; (vi) −*x*, −*y*+1, −*z*+1; (vii) −*x*+3/2, *y*−1/2, −*z*+1/2.

### Cation geometry (°)

**Table:**

Torsion angle	Value
O1A-C1A-C2A-C3A	-164.4 (2)
C1A-C2A-C3A-N1A	-62.9 (3)
	
O1B-C1B-C2B-C3B	171.4 (2)
C1B-C2B-C3B-N1B	59.7 (3)

### Anion geometry (Å, °)

**Table:**

Pb1-Br1	3.0589 (3)	Pb1-Br2	2.8952 (3)	Pb1-Br4	2.9477 (3)
Pb1-Br3	2.9230 (4)	Pb1-Br2^i^	3.2714 (2)	Pb1-Br4^ii^	3.0591 (3)
					
Br1-Pb1-Br2	88.11 (1)	Br2-Pb1-Br4	90.57 (1)	Br3-Pb1-Br2	103.14 (1)
Br1-Pb1-Br2^i^	80.90 (1)	Br2-Pb1-Br4^ii^	96.64 (1)	Br3-Pb1-Br2^i^	87.83 (1)
Br1-Pb1-Br4	87.68 (1)	Br2^i^-Pb1-Br4	90.75 (1)	Br3-Pb1-Br4	92.60 (1)
Br1-Pb1-Br4^ii^	89.76 (1)	Br2^i^-Pb1-Br4^ii^	81.63 (1)	Br3-Pb1-Br4^ii^	90.47 (1)
					
Pb1-Br2-Pb1^iii^	168.87 (1)	Pb1-Br4-Pb1^iv^	168.90 (1)		
Pb1-Br2^i^-Pb1^i^	168.87 (1)	Pb1-Br4^i^-Pb1^ii^	168.90 (1)		

Symmetry codes: (i) x-1, y, z; (ii) -x+1/2, y-1/2, -z+1/2; (iii) x+1, y, z; (iv) -x+1/2, y+1/2, -z+1/2.
